# An Experimental Investigation of the Mechanism of Hygrothermal Aging and Low-Velocity Impact Performance of Resin Matrix Composites

**DOI:** 10.3390/polym16111477

**Published:** 2024-05-23

**Authors:** Yuxuan Zhang, Shi Yan, Xin Wang, Yue Guan, Changmei Du, Tiancong Fan, Hanhua Li, Junjun Zhai

**Affiliations:** 1 Department of Engineering Mechanics, Harbin University of Science and Technology, Harbin 150000, China; 15682852313@163.com (Y.Z.); wangxiniiiiii@outlook.com (X.W.); 15155944384@163.com (Y.G.); 18306393902@163.com (C.D.); 2 Aulin College, Northeast Forestry University, Harbin 150000, China; m15774519625@163.com; 3 Department of Engineering Mechanics, Beijing Institute of Astronautical Systems Engineering, Beijing 100076, China; 13b918023@hit.edu.cn; 4 College of Aeronautics and Astronautics, North China Institute of Aerospace Engineering, Langfang 065000, China; junzhai726@nciae.edu.cn

**Keywords:** resin matrix composites, hygrothermal aging, three-dimensional braided composites, Fourier transform infrared spectroscopy (FT-IR), thermogravimetric analysis (TGA), low-velocity impact (LVI), C-scan, SEM

## Abstract

Resin matrix composites (RCs) have better thermal and chemical stability, so they are widely used in engineering fields. In this study, the aging process and mechanism of two different types of resin-based three-dimensional four-way braided composites (H15 and S15) under different hygrothermal aging conditions were studied. The effect of aging behavior on the mechanical properties of RCs was also studied. Three different aging conditions were studied: Case I, 40 °C Soak; Case II, 70 °C Soak; and Case III, 70 °C-85% relative humidity (RH). It was found that the hygroscopic behavior of RCs in the process of moisture-heat aging conforms to Fick’s second law. Higher temperatures and humidity lead to higher water absorption. The equilibrium hygroscopic content of H15 was 1.46% (Case II), and that of S15 was 2.51% (Case II). FT-IR revealed the different hygroscopic mechanisms of H15 and S15 in terms of aging behavior. On the whole, the infiltration behavior of water molecules is mainly exhibited in the process of wet and thermal aging. At the same time, the effect of the aging process on resin matrices was observed using SEM. It was found that the aging process led to the formation of microchannels on the substrate surface of S15, and the formation of these channels was the main reason for the better moisture absorption and lower mechanical strength of S15. At the same time, this study further found that temperature and oxygen content are the core influences on post-aging strength. The LVI experiment also showed that the structural changes and deterioration effects occurring after aging reduced the strength of the studied material.

## 1. Introduction

Compared with traditional two-dimensional laminates, three-dimensional braided composites (3D-BCs) have better integrity and higher specific strength, so they are widely used in the engineering field [1,2,3]. Among them, resin matrix composites are widely used in civil infrastructure facilities, offshore engineering, and other areas due to their better thermal and chemical stability, excellent fatigue resistance, and strong designability [4,5,6]. Hygrothermal environments are among the most common aging environments in the extensive service environments in which resin-based 3D-BCs are employed.

It has been proved that the hygrothermal environment in which composite materials are situated will adversely affect their properties [7,8,9,10]. Reports further point out that such adverse effects include reduced plasticization, a lower glass transition temperature (Tg), creep, increased stress relaxation, reduced mechanical strength, and a reduced elastic modulus [11,12,13,14,15,16,17]. Dao, B., et al. have also investigated the mechanism of hygrothermal aging. Some scholars believe that in the process of hygrothermal aging, the absorbed water may react with the unreacted groups in the structure of epoxy resin, thus affecting the structure of the material [18]. Apicella, A., et al. and Liu, Weiping, et al. believe that there are two main types of absorbed water molecules: “free water” and “bound water” [19,20]. Up to now, researchers’ descriptions of the water absorption behavior of resin matrix composites have been based on Fick’s law [21,22,23] and the Langmuir model [24,25]. It is generally believed that the resin matrix is the main factor in the process of hygrothermal aging. However, in fact, the aging process not only affects the resin matrix but also the fiber/resin interface [26,27]. The fiber/resin interface is an important factor determining the properties of composite materials, and the corresponding hygrothermal environment can reduce the bonding efficiency of the interface, leading to degradation. The deterioration phenomenon will further promote the diffusion of water molecules, resulting in more serious deterioration [28]. In addition, higher temperatures enhance the mobility of water molecules, which further accelerates the degradation rate of the resin base [29,30].

Abd Baghad and Khalil El Mabrouk [31] systematically studied the effects of porosity and aging behavior on the hygroscopic behavior, diffusion characteristics, thermomechanical properties, and mechanical properties of laminates. Their research shows that hygrothermal aging reduces the glass transition temperature (Tg), compression modulus, and shear strength of these materials. Pietro Aceti et al. [32] mainly reported on the influence of hygroscopic behavior on the mechanical properties of composites, focusing on summarizing the nature of damage occurring after the exhibition of hygroscopic behavior and the degradation mechanism leading to the change of properties. Lilla Mansour et al. [33] mainly reported on the effect of hygrothermal aging in different media on the mechanical properties of short-fiber/woven hybrid composite laminates. The results showed that aging time, medium, and temperature have significant effects on the mechanical properties of composites. Dandan Liao et al. [34] reported the aging behavior and aging mechanism of glass steel pipes under different aging conditions. In their study, an increase in ester base C-O bonds and a decrease in C–O–C and C–H bond strength during thermal oxidation were systematically demonstrated, thus demonstrating the water absorption and hydrolysis behavior of glass fibers during aging. Filip Vukovi et al. [35] simulated the aging process of a new epoxy resin/carbon fiber interface using molecular dynamics. Their simulations showed that the fiber–matrix interface is not susceptible to initial water entry by hygroscopic types and is not prone to flooding during these early stages of water aging. In contrast, water is preferentially absorbed through the matrix–water interface.

The materials used in this experiment are widely used in the aviation industry, and the service environment in which hygrothermal aging occurs has a crucial impact on the durability of these materials. At the same time, the structure of an aircraft is very vulnerable to the impact of foreign objects, resulting in some invisible damage, which will also have an important impact on the durability and water absorption of the structure. Up to now, although relevant studies have reported on the influence of the hygrothermal environment on the properties of resin matrix composites, there is still a lack of microanalyses in which multiple characterization methods are used to analyze the influence of complex aging environments on the mechanical properties of composite materials. There are few studies on the impact response of composite materials after aging, and no unified view has been developed [36]. Similarly, there are still few comparative studies on different resin matrix composites. In this project, two different kinds of resin-based 3D-BCs were designed to test hygrothermal aging in three different aging environments, namely, Case I (40 °C Soak), Case II (70 °C Soak), and Case III (70 °C-85% RH), and at three different aging times (500 h, 1000 h, and 2000 h). After that, an LVI test was conducted to analyze the influence of the aging process on mechanical properties. In the whole process, thermogravimetric analysis (TGA) and Fourier transform infrared spectroscopy (FT-IR) were used to analyze the hygroscopic behavior and chemical composition of the samples after aging. The surface of the matrix before and after aging was characterized using scanning electron microscopy (SEM). The effect of aging behavior on the structures and chemical compositions of the specimens was further analyzed. The influence of aging behavior on the mechanical properties of the specimens was verified in the LVI test. This work not only characterized the aging behavior of resin matrix composites in detail and analyzed their compositions but also provides an experimental basis for understanding and studying the corresponding aging mechanism. This work will be conducive to the further development of engineering applications and numerical simulations of resin matrix composites.

## 2. Experimental Procedure

### 2.1. Materials

**Figure 1:**
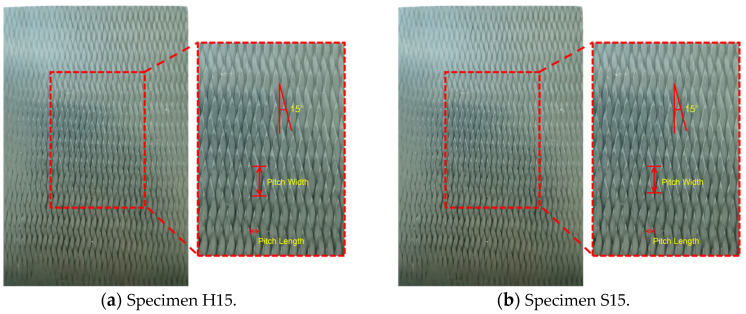
3D4d-BC specimens.

The material used in this experiment is 3D4d-braided composite (3D4d-BC) with a braided angle of 15°. The specific parameters are as follows: the thermosetting resin matrix had the epoxy resin matrix of model TDE-86, denoted as H15. TDE-86 is an alicyclic glycidyl ester type three-functional epoxy resin. Its glass conversion temperature (Tg) is about 219.5 °C, and it was cured using a single-component curing agent. The thermoplastic resin matrix used was double horse resin matrix 6428, denoted as S15. The glass conversion temperature of type 6428 resin is about 240 °C, and it was cured using a single-component curing agent. It was provided by Hubei Feilihua Quartz Glass Co., Ltd (Jingzhou, China). The preyarn of H15 and S15 uses Toray T700-SC-1000-50B carbon fiber. Due to errors in the manufacturing process, the statistical fiber volume content of all experimental materials ranged from 60.91% to 62.85%, with an average content of 62.02%. In this case, the yarn fineness is 800 tex, the braided angle is 15°, the pitch length is 41.0 ± 1.0 mm, and the pitch width is 11.0 ± 0.5 mm. The specific parameters are shown in Table 1. The dimensions of these materials are 120 mm × 80 mm × 5 mm, and the actual measurements have an error of ±0.5 mm, as shown in Figure 1.

### 2.2. Hygrothermal Aging Process

Three kinds of hygrothermal aging conditions were generated in this experiment. The three cases are Case I—40 °C Soak, Case II—70 °C Soak, and Case III—70 °C 85% relative humidity (RH). The aging times were 500 h, 1000 h, and 2000 h. Distilled water was used in the hygrothermal process. A constant temperature and humidity tester (JHY-H-150L, Xiamen, China) were employed. Two groups of parallel tests were conducted in each group, and two groups of specimens in normal-temperature environment (that is, non-aged) were set as the control group.

The sample-numbering rule used was “matrix type—braiding Angle—hygrothermal aging time—hygrothermal aging environment”, and “R” denotes room-temperature environment. For example, “H15-500-70 °C/85%RH” is an “H” matrix with a braided angle of 15° for 500 h of hygrothermal aging at 70 °C/85%RH. The experimental materials are shown in Table 2.

### 2.3. Gravimetric Measurement

The initial weights of each specimen were recorded as M0 before the hygrothermal aging process was induced. During the aging process, specimens were removed from the equipment at the same time every day and then dried, weighed, recorded, and returned to the equipment for further aging. The calculation formula of moisture absorption content is

(1) $$M_{t}=\frac{M_{i}-M_{i-1}}{M_{0}}\times 100\%$$

where “*M_i_*” is the average quality of the test part measured on day “*i*” (*i* = 1, 2, 3…), and “*M_t_*” is the hygroscopic content at time “*t*”.

### 2.4. FT-IR

In order to further understand the effect of the hygrothermal aging process on the compositions and structures of resin molecules, samples not subjected to an aging process (#1 and #11) and samples aged for 2000 h (#8, #9, #10, #18, #19, and #20) were analyzed using Fourier transform infrared spectroscopy (FT-IR). The middle part of each sample was ground to produce about 1 ml of powder. The powder was mixed with KBr powder and pressed into thin sheets for FT-IR analysis.

### 2.5. TGA

Thermo-gravimetric analysis of the specimens subjected to hygrothermal aging was performed using air purging at a rate of 50 mL/min via a METTLER TOLEDO TGA/DSC 3+ (Bern, Switzerland). At a heating rate of 10 K/min, “H15” was heated from 30 °C to 150 °C, and “S15” was heated to 200 °C.

### 2.6. LVI Test

**Figure 2:**
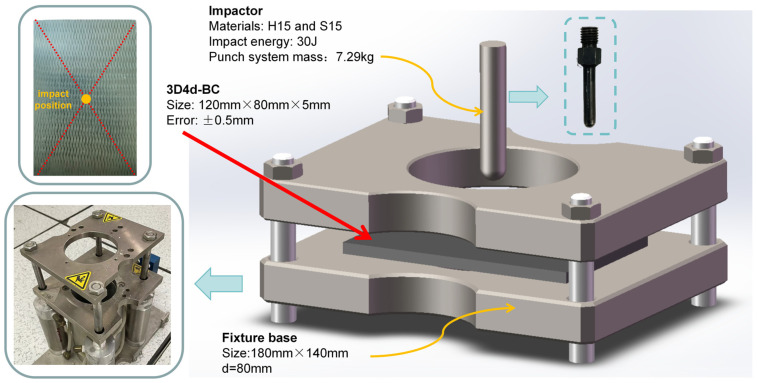
Schematic diagram of LVI test.

In order to study the effect of hygrothermal aging on impact performance, a low-velocity impact (LVI) test was carried out using 30 J of single-impact energy via a falling hammer machine (Instron-9250HV, Norwood, MA, USA). The impact position was the geometric center of the test piece. The single-headed ball punch used in the LVI experiment (mass of 0.145 kg) has a total impact platform mass of 7.29 kg. After the LVI test, the specimen was subjected to scanning acoustic microscopy (C-scan) via Sonoscan-D9500 (Santa Clara, CA, USA). The scanning area was set to 80 mm × 80 mm. A schematic diagram of the LVI test is shown in Figure 2.

### 2.7. SEM

The damage area of LVI specimens and the unaged resin structures were observed with a scanning electron microscope via TESCAN AMBER (TESCAN, Bragg, Czech) and SU5000 (Hitachi, Tokyo, Japan). The middle of each specimen was peeled to make a thin slice of about 5 mm × 5 mm that served as the sample for SEM testing. Due to the poor electrical conductivity of the tested materials, the materials’ surfaces needed to be treated with platinum plating in a vacuum environment to improve the electrical conductivity of the materials.

## 3. Results and Discussion

### 3.1. Hygrothermal Aging Process

In general, the process of polymer mass increase (the Fickian diffusion process) caused by water absorption at time t by the diffusion of a single free phase can be described by Formula (2) [37].

(2) $$\frac{M_{t}}{M_{m}}=1-\sum_{n=0}^{\infty }\frac{8}{{\left(2n+1\right)}^{2}\pi^{2}}\exp\left[-\frac{D{\left(2n+1\right)}^{2}\pi^{2}t}{4h^{2}}\right]$$

where “*M_t_*” represents the hygroscopic content at time “*t*”, “*M_m_*” is the equilibrium value of diffused water, “*D*” is the diffusion coefficient, and “*h*” is the thickness of the specimen.

**Figure 3:**
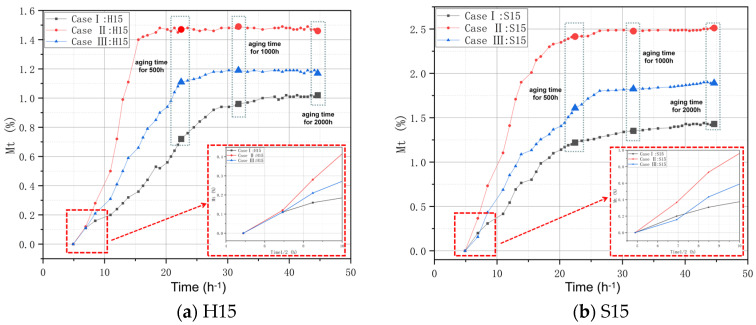
The square root relationships between moisture absorption and aging time of specimens in Case I, Case II, and Case III.

According to relevant studies, for the linear part of the water absorption curve (Figure 3), the diffusion coefficient “*D*” can be calculated according to the slope of the water absorption curve and the square root of time. In this state, *Dt*/*h*^2^ < 0.05, and Formula (2) can be simplified to Formula (3).

(3) $$\frac{M_{t}}{M_{m}}=\frac{4}{h}\sqrt{\frac{Dt}{\pi }}$$

When *Dt*/*h*^2^ > 0.05, Formula (2) can be simplified to Formula (4).

(4) $$\frac{M_{t}}{M_{m}}=1-\frac{8}{\pi^{2}}\exp\left(-\frac{Dt}{h^{2}}\pi^{2}\right)$$

For all samples subjected to an aging process, the moisture absorption (m) in the early stage and the square root of aging time showed a linear increase trend, indicating that the moisture absorption process followed Fick’s second law. The square root relationships between moisture absorption and aging time of the specimens in Case I, Case II, and Case III are shown in Figure 3. As shown in Figure 3, the equilibrium hygroscopic content and water diffusion rate of the two matrixes both adhere to the following order: Case II > Case III > Case I. Thus, the following conclusions can be drawn:

(1) Higher temperatures and higher humidity will facilitate a greater water absorption process. Specifically, when the final state is reached, the equilibrium hygroscopic content of Case II is 1.46% (H15) and 2.51% (S15). Under different temperatures and humidities, the equilibrium moisture absorption content of H15 increased by 0.44% and 43.14%, respectively. The equilibrium hygroscopic content of S15 increased by a maximum of 1.08% and a maximum of 75.59%.
(2) Compared with the “H” matrix, the “S” matrix has better water absorption under aging conditions. This indicates that for substrates with different chemical compositions, the hygrothermal aging condition has a certain influence on the chemical reactions and rates of in the aging process. This facet will be further analyzed in the “FT-IR analysis” Section.
(3) The water absorption process is faster in the early stage of aging. This is due to the fact that since the matrix is not completely dense and uniform, under the influence of capillary action, water molecules are attracted to the voids inside the material. Related studies have shown that at this stage, water molecules are almost uniform and stable in the interior of the material [38].

### 3.2. FT-IR Analysis

**Figure 4:**
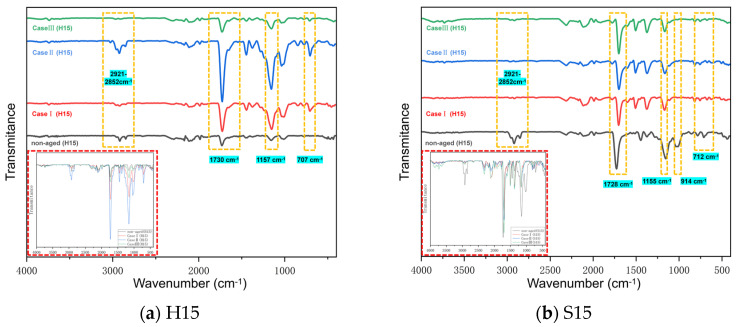
FT-IR spectra of Case I, Case II, and Case III under a hygrothermal aging time of 2000 h.

The effect of hygrothermal aging behavior on materials often occurs at the molecular scale, which is generally manifested as the change of chemical molecules [39]. Based on this, FT-IR analysis was performed for Case I, Case II, and Case III, and the results are shown in Figure 4.

The distributions of characteristic absorption bands in the FT-IR images of H15 (Figure 4a) and FT-IR images of S15 (Figure 4b) are basically the same. The hydrolysis of the resin matrix mainly occurs in the ester group (~1744 cm^−1^)/epoxy group (~914 cm^−1^). The oxidation behavior mainly occurs in methyl or methylene groups (~2920 cm^−1^ and ~2850 cm^−1^) between benzene rings. Therefore, by analyzing the change in peak strength of these major groups, we can analyze the mechanism of hygrothermal aging behavior.

With the aging of the two materials, the hydrolysis of the matrix also occurred. Using FT-IR, it was found that the reasons for the hydrolysis of the two substrates were different. Specifically, at 1730 cm^−1^, the hydroxyl peak strength of the epoxy ester group in H15 increased with the change in temperature (Case I and Case II) and decreased with the change in oxygen content (Case II and Case III). This indicates that there is a reaction between the water ester groups, which may be caused by the oxidation of the methylene bridge (–CH_2_^−^) (~2930 cm^−1^) in the epoxy resin during the aging process, generating the stretching vibration product of the C=O group in the ester group [40,41]. The peak intensity of S15 at 1730 cm^−1^ was almost unchanged, while the absorption band intensity at 915 m^−1^ was significantly reduced. This means that, at this time, S15 completes matrix curing hardening by consuming the unreacted epoxy group, promoting post-curing. It has been suggested in the literature that this change means that the resin base is completely cured after aging [42,43].

Similarly, oxidation behavior is ongoing during the aging process. Changes in the intensity of the characteristic absorption bands near 2918 cm^−1^ and 2849 cm^−1^ indicate the oxidation of methyl or C–H bonds on methylene between the two benzene rings [44]. This suggests that an increase in temperature will accelerate the rate of oxidation. It is also worth noting that the characteristic absorption band changes near 1157 cm^−1^ indicate that the C–O–C bonds in H15 and S15 are affected by the aging of the resin matrix [18,45,46,47].

Therefore, the infiltration of water molecules under the action of hygrothermal aging will further degrade the epoxy resin [48,49]. It has been reported that the degradation of resin is influenced by its own chemical composition, temperature, and pH [50]. From this, we determined that there are two main changes in the process of hygrothermal aging: one is the post-curing process. In this process, the crosslinking density increases, and the internal stress decreases [51]. Second, the resin is subject to the entry of water molecules during the aging process. The combination of these factors will further affect the mechanical properties of a specimen.

### 3.3. TGA Analysis

**Figure 5:**
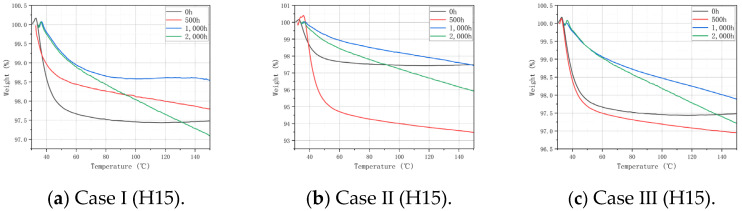
TGA curves of H15 in Case I, Case II, Case III, and the non-aged control.

**Figure 6:**
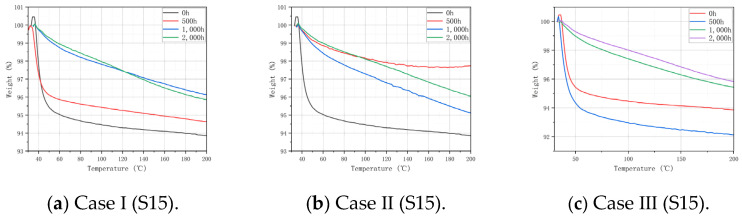
TGA curves of S15 in Case I, Case II, Case III, and the non-aged control.

TGA was performed for Case I, Case II, Case III, and the control group (non-aged specimens), and then the influence of different humidity and temperature conditions on the thermal stability of the matrix was analyzed. The TGA curves of H15 and S15 are shown in Figure 5 and Figure 6. The maximum mass loss statistics of S15 and H15 in TGA are shown in Table 3 and Table 4. On the whole, the thermo-gravimetric curves of H15 and S15 show the same changing trend: the greater the aging time, temperature, and oxygen content, the faster the weight decline, and the greater the impact on the thermal stability of the material.

It can be seen from Figure 5 and Figure 6 that after 500 h of the aging treatment, the weight of H15 and S15 in Case I and Case III decreases significantly at 50–60 °C, and the mass loss is basically the same as that in the non-aging state (Table 3). The lower mass loss in Case II indicates that temperature is an important factor affecting the thermal stability of the matrix. After 1000 h and 2000 h of the aging treatment, the temperature changed the most when the weight changed the most in the three cases, indicating that the thermal stability of the matrix was affected. According to the curve law shown in Figure 5 and Figure 6, it can be concluded that the thermal stability of S15 is higher than that of H15.

It should be noted that at the beginning of the curve, the weight percentage slightly exceeded 100% because the heating density of the gas around the crucible decreased after heating, and the buoyancy decreased, resulting in a certain weight gain. Such an experimental error does not affect the analysis and can be ignored.

### 3.4. LVI Test Analysis

**Figure 7:**
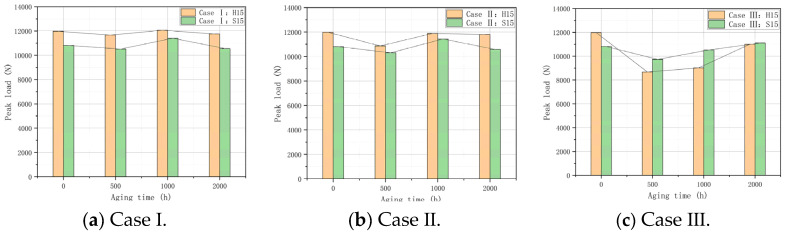
The relationship between peak load (H15 and S15) and aging time in Case I, Case II, and Case III.

An LVI test was carried out on all the aged specimens, and the relationship between peak load and aging time under three conditions was calculated, as shown in Figure 7. In general, the peak loads in the LVI test under different aging conditions were as follows: Case II > Case I > Case III (H15) and Case I > Case II > Case III (S15). Throughout the LVI test, we found that aging time is not the most critical factor affecting the degradation of material properties. The addition of oxygen during aging is an important reason for the deterioration of the impact resistance of the material. Specific analysis: In the whole LVI test, the peak load of H15 was higher than that of S15. Combined with the results of previous water and heat aging studies, H15 has better water absorption. It was determined that the higher water content provided the material with better impact resistance and toughness. The peak load of H15 is higher than that of S15 when it is non-aged. Therefore, the maximum variation of the two factors “temperature” and “humidity” under a single variable was calculated. The results show the following:

Under the condition of using only “temperature” as a variable (Case I and Case II), H15 decreased from 12,069.62 N to 10,865.73 N, and the peak load reduction rate was 9.97%, while S15 decreased from 11,433.35 N to 10,304.26 N, and the peak load reduction rate was 9.88%. With the increase in aging time, the peak load exhibited a “rising–falling” trend. This shows that temperature has a similar influence on the strength of the two substrates during the hygrothermal aging process.

Under the condition of only using “humidity” as a variable (Case II and Case III), H15 decreased from 12,069.62 N to 8673.10 N, and the peak load reduction rate was 28.14%, while S15 decreased from 11,406.32 N to 9734.90 N, and the peak load reduction rate was 14.65%. With the increase in aging time, the peak load showed a trend consisting of “great decreasing—upward decreasing”. This shows that the aerobic environment becomes a key factor in the deterioration of material properties during the aging process. At the same time, it shows that S15 has better thermal stability than H15.

**Figure 8:**
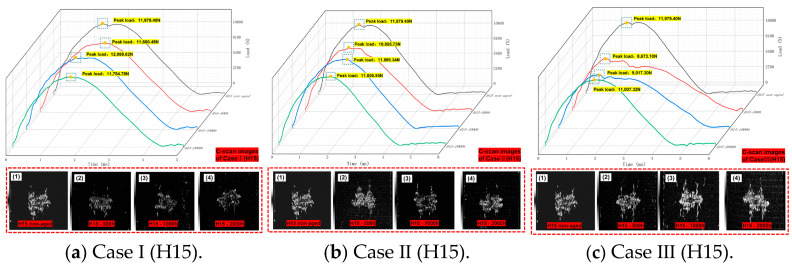
H15 “Time-Load” curves for Case I, Case II, Case III, and the non-aged control. In (**a**), **1**, **2**, **3**, and **4** correspond to C-scan images with aging time of 0 h, 500 h, 1000 h, and 2000 h in CaseI, respectively. In (**b**), **1**, **2**, **3**, and **4** correspond to C-scan images with aging time of 0 h, 500 h, 1000 h, and 2000 h in Case II respectively. In (**c**), **1**, **2**, **3**, and **4** correspond to C-scan images with aging time of 0 h, 500 h, 1000 h, and 2000 h under Case III conditions respectively.

**Figure 9:**
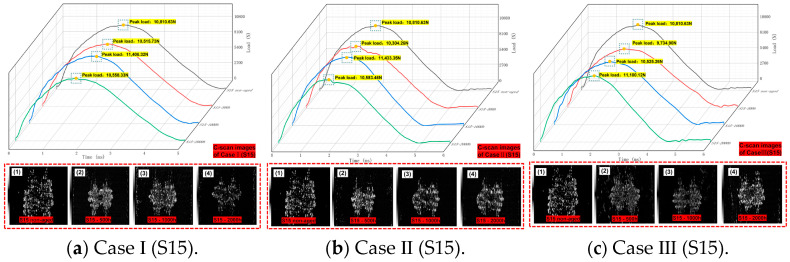
S15 “Time-Load” curves for Case I, Case II, Case III, and the non-aged control. In (**a**), **1**, **2**, **3**, and **4** correspond to C-scan images with aging time of 0 h, 500 h, 1000 h, and 2000 h in CaseI, respectively. In (**b**), **1**, **2**, **3**, and **4** correspond to C-scan images with aging time of 0 h, 500 h, 1000 h, and 2000 h in Case II respectively. In (**c**), **1**, **2**, **3**, and **4** correspond to C-scan images with aging time of 0 h, 500 h, 1000 h, and 2000 h under Case III conditions respectively.

The recorded “Time-Load” curve is shown in Figure 8 and Figure 9, summarizing the peak load and C-scan images in all cases (non-aged, Case I, Case II, and Case III). We found that the “Time-Load” curve as a whole presents three stages of “smooth ascending—reaching peak load—smooth descending”. At the same time, the overall impact response lengthens with the increase in hygrothermal aging time. This shows that with the increase in water absorption of the material, the toughness of the material will increase. After the LVI test, a C-scan was performed on all specimens, with an average depth of 0.15 mm. The depth was selected as the maximum peak value of the reflected wave, where the damage is the greatest, and the scanning depth is not consistent with a slight deviation. The white part in the C-scan images indicates the appearance of an internal damaged area, meaning that minor damage such as fiber and matrix debonding or fracturing has occurred. As the toughness of the material increased, its stiffness decreased, so the internal damage after an impact also increased. The proportion of damage area to scan area is shown in Table 5. According to statistics, after the same aging process, the ranking of internal damage performance is as follows: S15 > H15. At the same time, the damage area mainly exhibited the following behavior: with the impact point as the center, the damage extended to both sides along the weaving angle, showing an incomplete symmetrical “lung lobe” damage shape.

### 3.5. SEM Analysis

**Figure 10:**
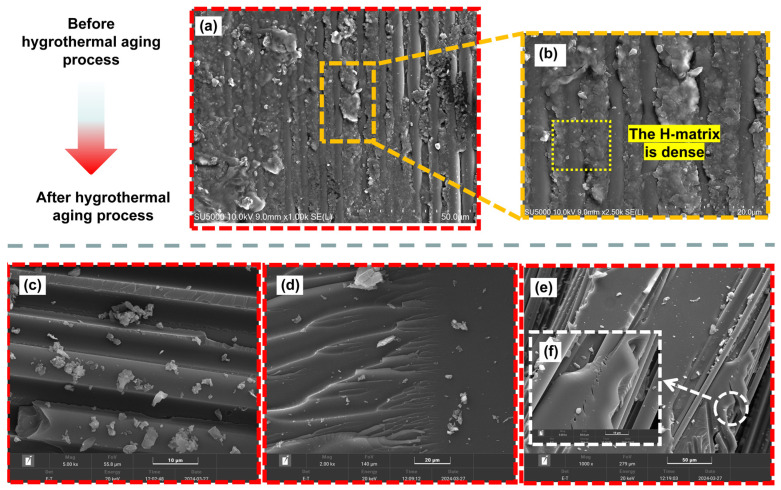
SEM images of H15 in Case I, Case II, Case III, and the non-aged control. (**a**,**b**) The matrix surface of H15 in a non-aged state is shown; (**c**) the matrix surface of H15 is shown for Case I (aging time 2000 h); (**d**) the matrix surface of H15 is shown for Case II (aging time 2000 h); (**e**,**f**) the matrix surface of H15 is shown for Case III (aging time 2000 h). Note: (**a**,**b**) were taken using a Hitachi SU5000, Japan.

**Figure 11:**
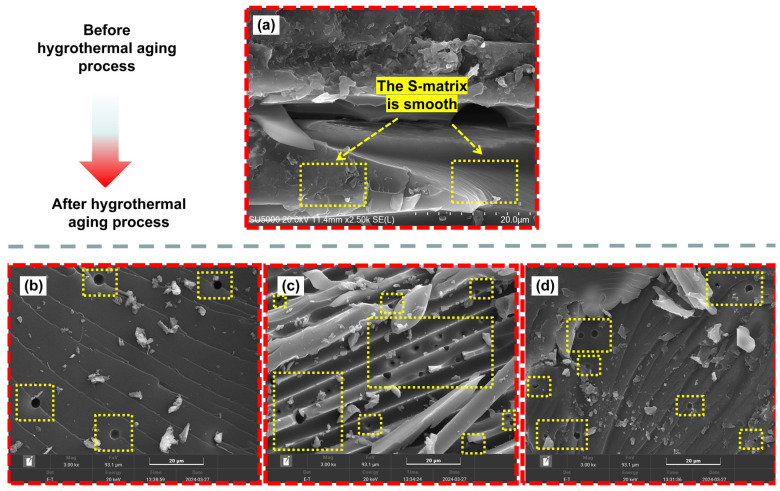
SEM images of S15 in Case I, Case II, Case III, and the non-aged control. (**a**) The matrix surface of S15 in a non-aged state is shown; (**b**) the matrix surface of S15 is shown for Case I (aging time 2000 h); (**c**) the matrix surface of S15 is shown for Case II (aging time 2000 h); (**d**) the matrix surface of S15 is shown for Case III (aging time 2000 h). Note: (**a**) was taken using a Hitachi SU5000, Japan, and the scale of the SEM images is 20 μm.

Figure 10 and Figure 11 show SEM images of the internal damage surface morphology of H15 and S15, respectively, after the LVI test was conducted. As shown in Figure 10a,b and Figure 11a, a large number of particles were present on the surface of the non-aged specimen due to polymer degradation. At the same time, the surface of the matrix of the non-aged specimen was relatively smooth. With the increase in aging time, it was generally observed that the longer the aging time and the higher the temperature, the more micro cracks and nodules. This indicates the deterioration and decomposition of the matrix. Specifically, the matrix of H15 does not exhibit small holes. However, on S15, the number of small holes increased as the aging time and temperature increased (Figure 11b–d). These micrometer-scale pores are proof that water molecules form microchannels inside the matrix during water absorption. The appearance of these channels indicates that the resin molecules induce oxidative degradation during the aging process, resulting in the entry of water molecules that produce microchannels. With the increase in temperature and the change in oxygen content, the number of small holes in the unit area also changes. Similarly, the presence of these microchannels is the main structural reason for the higher equilibrium hygroscopic content and lower strength of S15.

## 4. Conclusions

In this paper, the effects of temperature, humidity, and aging time on the structures and impact properties of resin-based 3D-BCs under different hygrothermal aging conditions (Case I, Case II, and Case III) were studied. The relevant research results reveal the following:

(1) The hygroscopic behavior in the process of aging under moist and hot conditions conforms to Fick’s second law. Higher temperatures and humidity lead to greater water absorption. For this study, the equilibrium hygroscopic content and water molecular diffusion rate of H15 and S15 corresponded to the following order: Case II > Case III > Case I. At the same time, this study shows that the maximum equilibrium moisture absorption content of H15 (Case II) was 1.46%, and the maximum equilibrium moisture absorption content of S15 (Case II) was 2.51%.
(2) By using FT-IR and TGA characterization methods, it was found that there are two main changes in the process of aging under moist and hot conditions: the crosslinking density increases and the internal stress decreases during the post-curing process. The infiltration of water molecules further degrades the epoxy resin. The effect of oxygen content on H15 is greater than that on S15. At the same time, the increase in temperature is also the cause of accelerated oxidation. The oxygen in the environment is an important factor affecting the process of hygrothermal aging. Due to the above reasons, aging treatment will further reduce the strength of the studied specimen. TGA revealed that aging time, temperature, and oxygen content are important factors affecting thermal stability. In this study, the thermal stability of S15 was higher than that of H15.
(3) Water molecules cause reversible and irreversible degradation mechanisms in polymers and composites, for example, swelling; molecular structure degradation; interruption of hydrogen bonds in polymers; increased interfacial degradation; increased stiffness; changes in mechanical properties, resulting in increased mobility of macromolecules; decreased Tg; and pseudo-ductility. At the same time, the combined influence of water molecules and temperature will also produce a synergistic effect, promoting plasticization, molecular degradation, post-polymerization reactions, etc., thus forming a network with different crosslinking densities through the formation of false crosslinking with multiple hydrogen bonds. Water molecules can also react with hydroxyl groups and thus form weak hydrogen bonds, resulting in the emergence of hydrophilic groups in epoxy resins, which, in turn, act as plasticizers.
(4) An LVI test and SEM were used to further explain the influence of hygrothermal aging on the impact properties of the studied materials. This work shows that aging time is not the core factor affecting the strength properties of these materials. The content of oxygen in the process of hygrothermal aging is the core factor that directly causes these materials’ strength to decrease. The SEM images also further explain the structural changes and degradation in the aging process at the microscopic level.

This work will be helpful for further studying the influence of the mechanical properties of resin matrix composites in hygrothermal environments. At the same time, it also provides an experimental research foundation for relevant numerical simulation work and lays an experimental foundation for the safe application of resin matrix composites in engineering.

## Figures and Tables

**Table 1:** Main parameters of test materials.

Type	Properties	
Carbon fiber	TORAY T700-SC-12000-50B	
Matrix	TDE-86-thermosetting resin matrix, epoxy resin matrix, (H)	Tensile strength: 54.69 MPa Tensile modulus: 4.10 Gpa Elongation at break: 1.99% Bending strength: 120.70 MPa
	6428-Thermoplastic resin matrix, bismaleimide resin matrix, (S)	Tensile strength: 57.2 MPa Tensile modulus: 2.5 Gpa Elongation at break: 2.5% Bending strength: 81.9 MPa
Fabric structure	Three-dimensional, four-directional	
Braided Angle	15°	
Yarn fineness	800 tex	
Pitch length	41.0 ± 1.0 mm	
Pitch width	11.0 ± 0.5 mm	
Overall fiber volume content	(60 ± 3)%	
Fibre filling factor	75%	

**Table 2:** Material numbering.

Number	Matrix of “H”	Number	Matrix of “S”
#1	H15-R	#11	S15-R
#2	H15-500-40 °C	#12	S15-500-40 °C
#3	H15-500-70 °C	#13	S15-500-70 °C
#4	H15-500-70 °C/85%RH	#14	S15-500-70 °C/85%RH
#5	H15-1000-40 °C	#15	S15-1000-40 °C
#6	H15-1000-70 °C	#16	S15-1000-70 °C
#7	H15-1000-70 °C/85%RH	#17	S15-1000-70 °C/85%RH
#8	H15-2000-40 °C	#18	S15-2000-40 °C
#9	H15-2000-70 °C	#19	S15-2000-70 °C
#10	H15-2000-70 °C/85%RH	#20	S15-2000-70 °C/85%RH

**Table 3:** Maximum weight loss rate of H15 in Case I, Case II, Case III, and the non-aged control.

Aging Time		0 h	500 h	1000 h	2000 h
Maximum weight loss rate (%)	Case I	2.52%	2.20%	1.49%	2.96%
	Case II	2.52%	6.51%	2.60%	4.16%
	Case III	2.52%	3.05%	2.16%	2.84%

**Table 4:** Maximum weight loss rate of S15 in Case I, Case II, Case III, and the non-aged control.

Aging Time		0 h	500 h	1000 h	2000 h
Maximum weight loss rate (%)	Case I	6.16%	5.37%	3.91%	4.23%
	Case II	6.16%	2.27%	4.97%	4.05%
	Case III	6.16%	7.88%	4.66%	4.29%

**Table 5:** The ratio of the internal damage area of the specimens to the scanning area after impact under hygrothermal aging.

Number	Damage Area Proportion (%)	Number	Damage Area Proportion (%)
#1	9.55	#11	11.59
#2	9.86	#12	14.77
#3	10.00	#13	15.45
#4	11.26	#14	18.18
#5	11.36	#15	14.95
#6	11.14	#16	15.91
#7	12.27	#17	18.86
#8	12.52	#18	15.94
#9	13.45	#19	19.45
#10	14.59	#20	21.57

## Data Availability

Data are contained within the article.
